# Predict Suitable Restoration Areas for Typical Vegetation Restoration Species on the Qinghai‐Tibetan Plateau Based on MaxEnt

**DOI:** 10.1002/ece3.73857

**Published:** 2026-06-19

**Authors:** Ying Yang, Chen‐Di Wang, Yan‐Gang Yin, Dong Han, Yue Zhong, Bo Wang, Guo‐Ying Zhou

**Affiliations:** ^1^ Sichuan Zoige Alpine Wetland Ecosystem National Observation and Research Station Southwest Minzu University Chengdu China; ^2^ College of Agriculture and Animal Husbandry Qinghai University Xining China; ^3^ Qinghai Traffic Construction Management Co., Ltd. Xining China; ^4^ Northwest Institute of Plateau Biology, Chinese Academy of Sciences Xining People's Republic of China

**Keywords:** climate warming, MaxEnt, niche breadth, niche overlap, species selection, vegetation restoration

## Abstract

Under global warming, the Qinghai‐Tibetan Plateau (QTP) ecosystem faces severe threats, and vegetation restoration is critical for ecological rehabilitation. Unlike previous studies focusing on endangered or invasive species, this research innovatively selects six typical vegetation restoration species ( *Poa pratensis* , *Poa araratica*, *Poa pratensis* var. *anceps*, *Festuca sinensis*, *Elymus nutans*, *Lolium perenne* ) and integrates species distribution modeling (MaxEnt), centroid migration analysis, and niche theory to systematically evaluate their restoration potential under current and future climate scenarios—advancing beyond conventional single‐species distribution predictions. Key findings: (1) Elevation (alt) and annual precipitation (bio12) are the dominant factors determining species distribution, with their combined contribution rates to the six species ranging from 46.6% ( *L. perenne* ) to 81.7% ( *F. sinensis* ), providing precise indicators for site selection in restoration. (2) Except for *L. perenne* , high‐suitability zones of other species concentrate in the northeastern QTP, with *E. nutans* having the largest suitable area (80.73 × 10^4^ km^2^, 61% of total QTP area). Under climate warming, 80% of species (e.g., *P. araratica*, *E. nutans* ) show significant habitat expansion (e.g., *E. nutans* moderate/high suitability zones increase by 90.79%/111.66% under RCP8.5 2070s) and an overall westward migration trend (e.g., *F. sinensis* migrates 246.51 km southwestward under RCP8.5). (3) *E. nutans* exhibits the widest niche breadth (B_1_ = 0.156, strongest adaptability) but high niche overlap with other species (e.g., *P. araratica* vs. *P. pratensis* , *D* = 0.75), indicating a potential competition risk requiring field validation when mixed‐sown in resource‐limited areas. This study applies a multidimensional assessment framework that integrates climate response, spatial migration, and niche interaction to QTP vegetation restoration, providing a scientific basis for species selection and configuration with important practical implications for climate‐resilient ecological restoration.

## Graphical abstract

It was first used in the QTP vegetation restoration species, mainly to provide a basis for species selection in different regions of the QTP now and in the future. Secondly, it calculates the ecological niche overlap and niche breadth to avoid the risk of interspecies competition, providing a basis for the combination of species. By bridging species distribution modeling, niche theory, and practical restoration ecology, we provided a basis for the selection and combination of species for vegetation restoration in different regions using QTP.

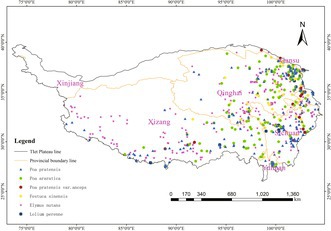

## 1 Introduction

The Qinghai‐Tibetan Plateau (QTP) is known as the “roof of the world” and the “third pole”. It is the highest and largest plateau region on the planet, and at the same time plays an important regulatory role in the global climate and ecosystem (Favre et al. 2015). The QTP has a unique geography and complex climatic conditions, and therefore is rich in biodiversity, and plays an important role in maintaining the global climate balance, supplying water resources, and maintaining ecological security (Favre et al. 2015; Liu et al. 2015; Lu and Guo 2014). But the ecosystem of the QTP is extremely fragile. In recent years, the QTP has been affected by climate change, overgrazing, agricultural expansion, and urbanization, leading to a decline in vegetation cover, increased soil erosion, and loss of biodiversity. Under the condition of global warming, the rate of temperature increase in the QTP is significantly higher than the global average, posing a severe threat to the ecosystem of the QTP (Kuang and Jiao 2016; Yao 2019; Ying et al. 2024; Zhong et al. 2019; Zou et al. 2018). Therefore, it is urgently necessary to carry out ecological restoration of the QTP; ecological restoration is not only important for the maintenance of the ecological balance of the plateau, but also has an important influence on the protection of the global ecosystem.

Vegetation restoration is a primary measure of ecological restoration and is widely applied in ecological recovery efforts in the QTP. It mainly involves the planting and arrangement of plants to restore and reconstruct damaged ecosystems (Duan et al. 2024; Li et al. 2025). Vegetation restoration plays a crucial role in the ecological environment of the plateau, as it can improve soil quality. Long‐term vegetation restoration can increase soil organic carbon (SOC) content (Gong et al. 2017) and enhance soil nutrient content, such as increasing total nitrogen (TN) and available nutrients (N, P, K) (Qi et al. 2018). Additionally, vegetation restoration can reduce soil erodibility. In the Loess Plateau, as the duration of vegetation restoration increases, the degree of soil and water loss shows a downward trend (Liu et al. 2025). Consequently, vegetation restoration is vital for maintaining the ecological balance of the QTP.

When conducting vegetation restoration, the selection of restoration species is very important. Only by choosing the right species for planting and matching can the role of vegetation restoration be better played. If the species selected are not suitable for local climatic conditions, vegetation restoration will not achieve the expected results. Moreover, due to the unique geographical environment and complex climatic conditions of the QTP, different regions of the QTP have different environmental conditions and suitable species are different. It is important to select plants that are suitable for local climatic conditions, in order to greatly improve the efficiency of vegetation restoration while reducing subsequent maintenance costs (Gao et al. 2019; Liu et al. 2022). Predicting and analyzing the distribution of suitable habitats for different plants in the context of a warming climate and the influencing factors is of great significance for the selection of species in different regions for vegetation restoration. Under the special environment of the cold and high altitude of the QTP, the growth of some vegetation is limited, and the growth period and plant height are lower than average. Therefore, it is suitable to select highly suitable species under these conditions, which can effectively restore the ecosystem service function. It is worth noting that if the species selected has great adaptability but is not native, it can lead to species invasion, affect the growth of native species, reduce biodiversity and lead to more serious consequences. Therefore, we chose to study the typical local restoration species *P. pratensis* , *P. araratica*, *P. pratensis* var. *anceps*, *F. sinensis* , *E. nutans* and *L. perenne* (Table 1), which have often been used in previous studies on vegetation restoration on the QTP. Predicting and analyzing the distribution of these plants' suitable habitats and the influencing factors in the context of a warming climate can provide a scientific basis for selecting species for revegetation in different areas of the QTP, and is of great scientific and practical significance for the ecological restoration of the QTP.

**TABLE 1:** Basic information and effective distribution points of six typical vegetation restoration species on the QTP.

Abbreviated	Species	Family name	Order name
PP	*Poa pratensis*	Gramineae	Poales
PA	*Poa araratica* (Basionym:*Poa crymophila*)	Gramineae	Poales
PPA	*Poa pratensis* var. *anceps*	Gramineae	Poales
FS	*Festuca sinensis*	Gramineae	Poales
EN	*Elymus nutans*	Gramineae	Poales
LP	*Lolium perenne*	Gramineae	Poales

Species Distribution Models (SDMs) are often used to predict suitable areas for species (Elith et al. 2011). They assess how species adapt to different climatic factors. Among various species distribution models, the Maximum Entropy (MaxEnt) model has high predictive accuracy and strong flexibility (Kaky et al. 2020; Merow et al. 2013; Wisz et al. 2008). It is widely used for predicting suitable habitats for species. In ecological studies of the QTP, the MaxEnt model has been successfully applied to predict suitable areas for various plants and animals, such as *Saussurea* (Zhao et al. 2024), endangered birds (Li et al. 2023), *Stipa purpurea* (Ma et al. 2022), and *Nardostachys jatamansi* (Wen et al. 2022).

When selecting suitable vegetation restoration species for different regions of the QTP, the concepts of niche breadth and niche overlap are two important reference indicators. The concept of ecological niche is used to explain the spatial distribution of species in their living environment and their utilization of resources. Niche breadth responds to a species' ability to adapt to its environment and use resources (Chase and Leibold 2003; Peterson et al. 2012). Species with broader niche breadths exhibit greater environmental adaptability. Thus, selecting such species for vegetation restoration can significantly improve its success rate. In regions with harsh climatic conditions, prioritizing species with high niche breadths is particularly advantageous for effective ecosystem recovery (Cai et al. 2021; Evans and Jacquemyn 2022). Niche overlap refers to the similarity in resource use or environmental requirements between two or more species coexisting in the same habitat. High niche overlap between species indicates highly similar ecological demands. In resource‐limited environments, such species may struggle to coexist long‐term due to competition for identical resources (Baltzer et al. 2007; Brown 1984). Analyzing niche overlap among species helps identify potential competitive interactions in vegetation restoration projects, thereby improving predictions of their coexistence potential in shared environments. This strategy prevents ecological conflicts from inappropriate species combinations, thereby enhancing restoration effectiveness (Chen et al. 2024).

Climate warming is driving significant shifts in species distributions worldwide, particularly in sensitive alpine ecosystems like the QTP. The QTP is warming at twice the global average rate (0.3°C–0.5°C per decade), leading to profound changes in thermal regimes, precipitation patterns, and permafrost dynamics (Lee et al. 2023). Cold‐adapted species migrate to higher elevations to maintain suitable temperatures; changes in precipitation patterns drive species to migrate toward regions with abundant water resources; and permafrost thaw caused by climate warming also reduces habitat suitability in low‐elevation areas (Guo et al. 2018; Wu et al. 2025; Zhao et al. 2024). Understanding these mechanistic links is critical for predicting species responses to climate change and developing effective vegetation restoration strategies.

Over the past five years, niche models for plateau vegetation restoration have evolved beyond predictions based on single algorithms or single species toward integrated approaches combining multiple algorithms, species, and scenarios. On the QTP, a spatial sliding window‐based Similar‐Habitat‐Based Vegetation Restoration Potential (SHB‐VRP) model couples sliding‐window maximum‐EVI extraction with niche‐similarity testing to separate policy‐driven greening from climate‐driven potential, providing grid‐scale restoration benchmarks for 2000–2020 (Yang et al. 2025). At the species level, Huang et al. (2024) combined MaxEnt with niche‐overlap metrics to forecast the future range of *Cotoneaster multiflorus* under CMIP6 scenarios and explicitly warned that high overlap with native shrubs may trigger competitive exclusion, highlighting the necessity of evaluating interspecific niche overlap before mix‐planting. Luo et al. (2024) employed the MaxEnt model to simultaneously evaluate 12 common tree, shrub and herb restoration species on the Loess Plateau, demonstrating that integrating climatic, edaphic and socio‐economic variables can explicitly map planting suitability and guide the vertical structuring of mixed vegetation layers. Guo et al. (2023) employed an ensemble‐SDM approach (GLM, GAM, RF, BRT and MaxEnt) to hindcast and forecast dominant woody species on the Loess Plateau, revealing that moderate warming could enlarge overall suitable area but that westward and upward centroid shifts will require seed‐matching and assisted migration in future restoration projects. Despite this progress, MaxEnt has not yet been integrated with classical niche theory (niche breadth and overlap metrics) to screen multiple restoration species simultaneously on the QTP.

The study employs the MaxEnt model to predict the suitable habitat of typical vegetation restoration species in the QTP for the first time. It analyzes the main environmental factors influencing the distribution of these species and calculates niche overlap and niche breadth. The objectives are: (1) to analyze the main environmental factors affecting the six typical vegetation restoration species in the QTP; (2) to predict that these species change in different temporal fitness regions in different climatic contexts; (3) to assess the niche breadth and niche overlap of these six species; and (4) to provide a scientific theoretical basis for species selection and combination in vegetation restoration in different regions of QTP.

## 2 Materials and Methods

### 2.1 Occurrence Data and Processing

**FIGURE 1:**
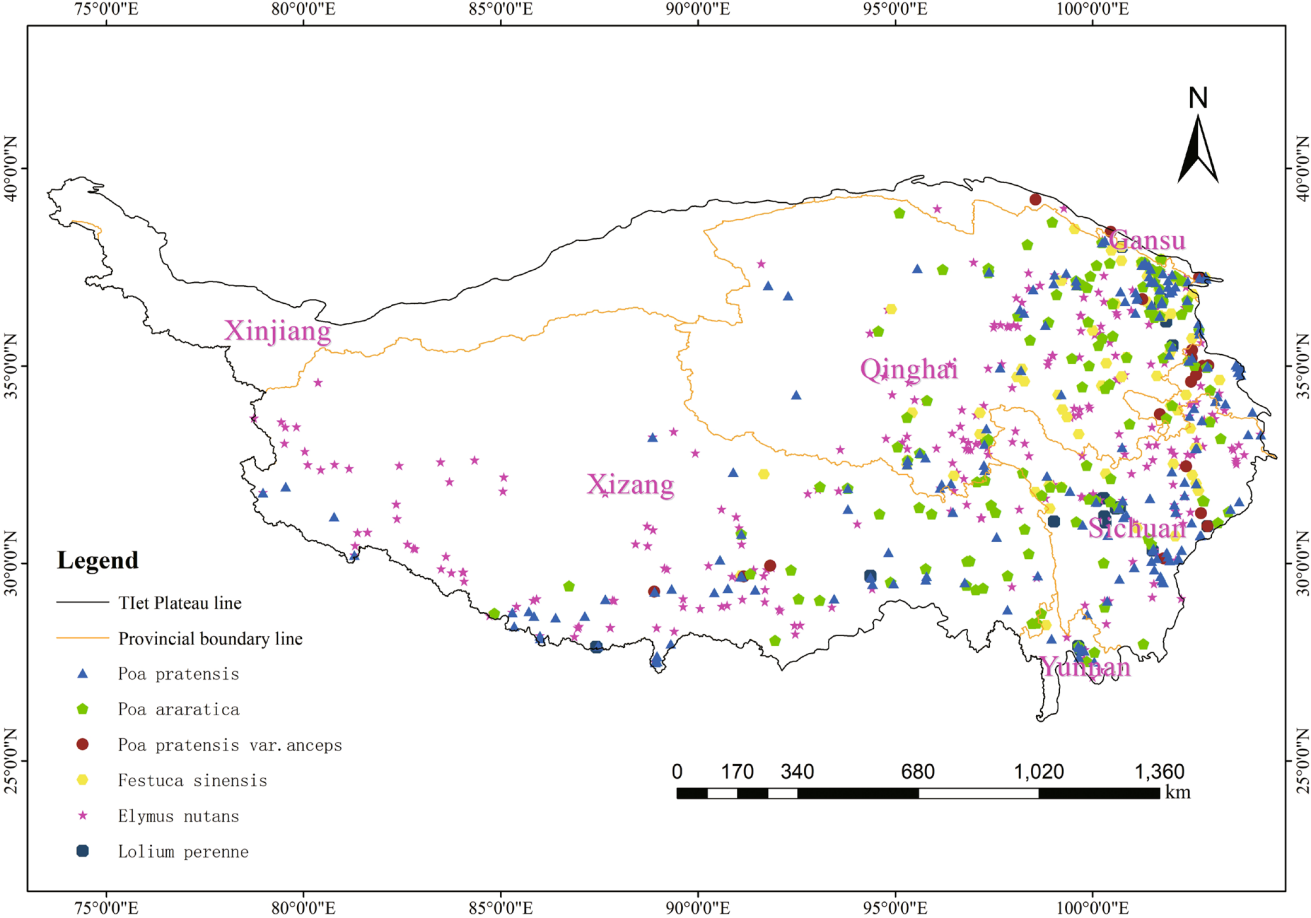
Current distribution points of species for vegetation restoration on the QTP.

We chose six typical vegetation restoration species in the QTP, including *Poa pratensis* , *Poa araratica (Basionym:Poa crymophila)*, *Poa pratensis* var. *anceps*, *Festuca sinensis*, *Elymus nutans*, and *Lolium perenne* , as our research subjects, with specimen photographs of these species presented in Figure S1. Distribution data were obtained from four sources: China Digital Herbarium (http://www.cvh.ac.cn), Teaching Specimen Resource Sharing Platform (http://mnh.scu.edu.cn), NSII China National Specimen Resource Platform (http://www.nsii.org.cn), and our field investigation. Strict data cleaning criteria were applied to ensure data quality: duplicate records with identical geographic coordinates (latitude/longitude to 6 decimal places) were retained only once, records outside the QTP administrative boundary or with ambiguous location information such as only county‐level labels without precise coordinates were excluded, and to avoid overfitting caused by clustered points, we used the “thin by grid” function in ENMTools with a 1 km × 1 km grid resolution, retaining only one occurrence point per grid cell (Li et al. 2023; Zhao et al. 2024). The finalized distribution datasets were stored in CSV format for subsequent MaxEnt analysis, species occurrence distribution maps were generated using ArcGIS (version 10.8) as shown in Figure 1, and detailed biological information for the six species are summarized in Table 1.

### 2.2 Acquisition of Climate Data and Environmental Variables

A total of 28 environmental factors were selected, including 19 bioclimatic factors, 1 topographic factor, and 8 soil factors (Table S1). The bioclimatic and topographic factor data were sourced from WorldClim (http://www.worldclim.org/) (1970–2000), with a resolution of 30 arcseconds (1 km × 1 km). Future climate factor data were obtained using CCSM4 climate change data, selecting two future climate datasets (2050s and 2070s) based on the greenhouse gas emission scenarios (Representative Concentration Pathways, RCPs) RCP2.6, RCP6.0, and RCP8.5, as published by the Intergovernmental Panel on Climate Change (IPCC)‐CMIP5. These data were sourced from the World Climate Database (https://www.sciencebase.gov/catalog/), with a resolution of 30 arcseconds. Soil factor data were obtained from the National Tibetan Plateau Data Center (https://data.tpdc.ac.cn/), where the HWSD China soil data were downloaded and resampled to a resolution of 30 arcseconds. All data were converted to ASC format for further use (Yang and Huang 2021).

**FIGURE 2:**
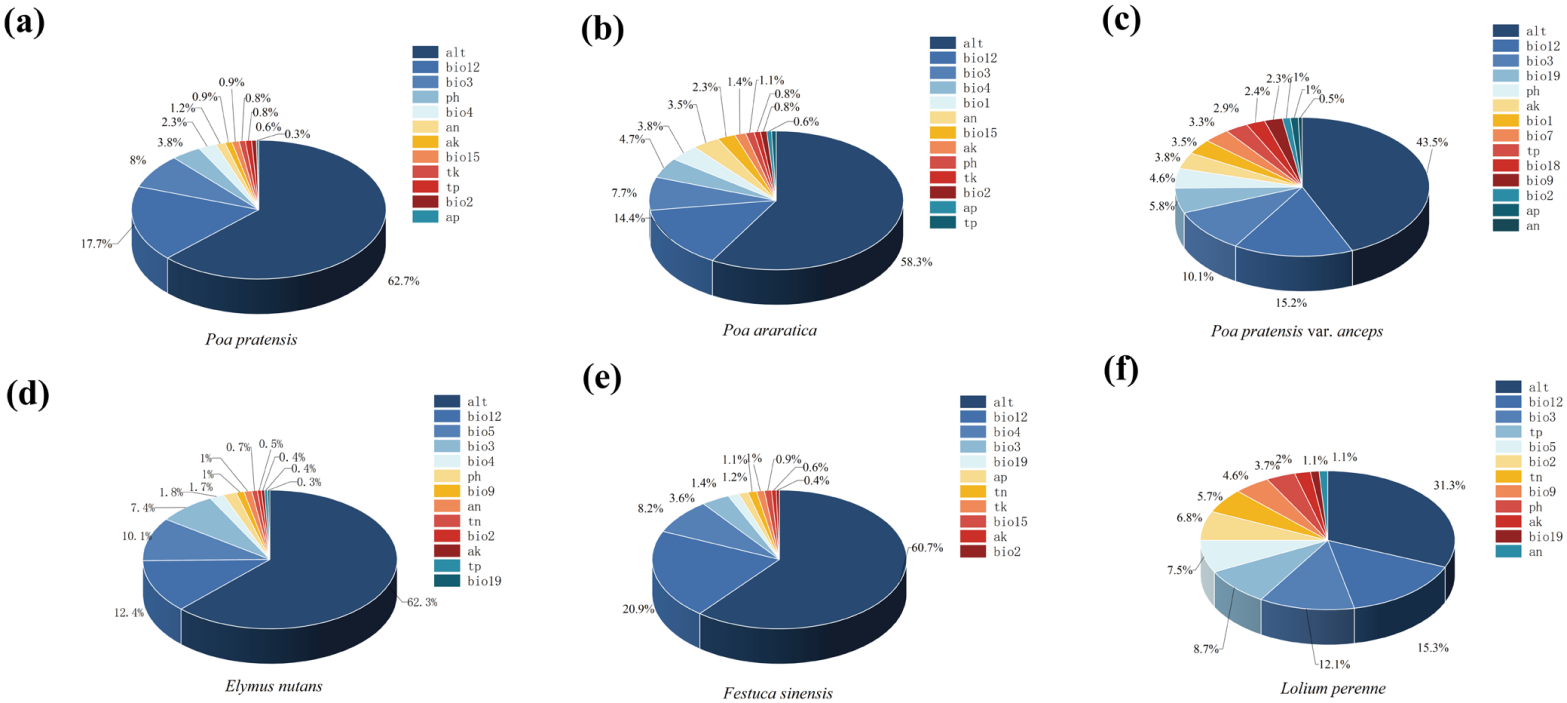
Contribution rates of environmental factors for six typical vegetation restoration species in the QTP.

To avoid inaccuracies in model predictions caused by the autocorrelation among various environmental factors, we implemented a two‐step screening process: Spatial subset extraction: We used ArcGIS 10.8 to clip all global environmental datasets to the QTP boundary, ensuring only regionally relevant data were used. Multicollinearity reduction: We calculated Pearson correlation coefficients between all pairs of environmental factors using ENMTools. For variable pairs with |*r*| > 0.8, we conducted a preliminary MaxEnt run to estimate each variable's relative contribution to the species distribution model (De Marco and Nóbrega 2018; Fu et al. 2022). The variable with the highest contribution rate was retained, while correlated counterparts were excluded to eliminate redundancy. Variables with |*r*| ≤ 0.8 were directly retained. After multiple rounds of screening, the number of final environmental factors selected for each species was: 13 for *P. pratensis* , 11 for *P. araratica*, 12 for *F. sinensis* , 13 for *P. pratensis* var. *anceps*, 13 for *E. nutans* , and 13 for *L. perenne* (Figure 2).

### 2.3 MaxEnt Model

We used MaxEnt to predict the geographic distribution patterns of plants under different climate scenarios across various periods. First, we set up the Java environment. Then, we imported the distribution data of the six typical vegetation restoration species and climate factor data for three periods (current, 2050s, and 2070s) into MaxEnt version 3.4.4. When building the prediction model, we allocated 25% of the data for model testing, selected the Jackknife method, and performed 10 repetitions, with a replicate run type of Bootstrap, set the regularization multiplier to 1.0 (default), specified the output format as Logistic, checked the options to make predictions of species distribution and do jackknife, set the maximum number of iterations to 5000 and convergence threshold to 0.00001 (default), and applied the 10 percentile training presence threshold rule to distinguish suitable from unsuitable areas. To ensure robustness and reduce stochasticity in the final maps, the logistic outputs from the 10 replicates were averaged before mapping. After establishing the model, we evaluated its accuracy using the ROC curve and the True Skill Statistic (TSS). The Area Under the Curve (AUC) value, which represents the area enclosed by the ROC curve and the horizontal axis, ranges from 0 to 1, with values closer to 1 indicating higher model accuracy (specifically, 0.5–0.7 indicates low accuracy, 0.7–0.9 indicates moderate accuracy, and 0.9–1 indicates very high accuracy). Additionally, the TSS, calculated as sensitivity plus specificity minus 1, provides a complementary measure of model performance that is independent of prevalence and ranges from −1 to 1. A TSS value closer to 1 indicates better model discrimination ability, with values ≤ 0.4 indicating poor performance, 0.4–0.7 indicating moderate performance, 0.7–0.8 indicating good performance, and > 0.8 indicating excellent performance (Fu et al. 2023).

The values of species growth suitability simulated by MaxEnt range from 0 to 1, with values closer to 1 indicating higher suitability for the species in that area. Based on the average values obtained from 10 repetitions of the MaxEnt model, we find the threshold (10 percentile training presence Logistic threshold) in “maxentResults” to separate unsuitable areas from suitable areas. In ArcGIS 10.8, we use the reclassification tool in the “Spatial Analyst” tools within “ArcToolbox” to classify the suitable distribution of each plant into four categories: areas below the threshold are classified as unsuitable, while areas above the threshold are divided into three equal parts, corresponding to low, medium, and high suitability, and we calculate the area of each suitable zone.

### 2.4 Calculation Niche Breadth and Niche Overlap

We used ENMTools v1.4 (http://purl.oclc.org/enmtools) to calculate the niche width of six typical vegetation recovery species on the QTP and the niche overlap among these species (Phillips et al. 2006; Warren et al. 2010). As a classic metric for quantifying niche breadth, Levins' Index evaluates the uniformity of a species' distribution across environmental resource states to reflect its resource utilization range. For cross‐species comparability, ENMTools outputs standardized Levins Index values (B1 and B2), which normalize the index to a fixed range of [0, 1]—values closer to 1 indicate a wider niche breadth. The standardized Levins Index is calculated as follows:

(1) $$B_{\mathrm{std}}=\frac{B-1}{n-1}$$

where (*n*) is the total number of environmental resource categories defined by the selected factors. For niche overlap, the software calculates two indices: Schoener's *D* (*D*) and Hellinger‐based *I* (*I*), where values closer to 1 indicate a higher degree of niche overlap.

### 2.5 Distribution Barycenter Migration

We used the Species Distribution Model (SDM) toolbox in ArcGIS software to calculate the geographic centers of six typical vegetation restoration species on the QTP under current and future scenarios. The coordinates of these distribution centers were determined using ArcGIS software, and migration trends were analyzed.

## 3 Results

### 3.1 Accuracy of the MaxEnt Models

**FIGURE 3:**
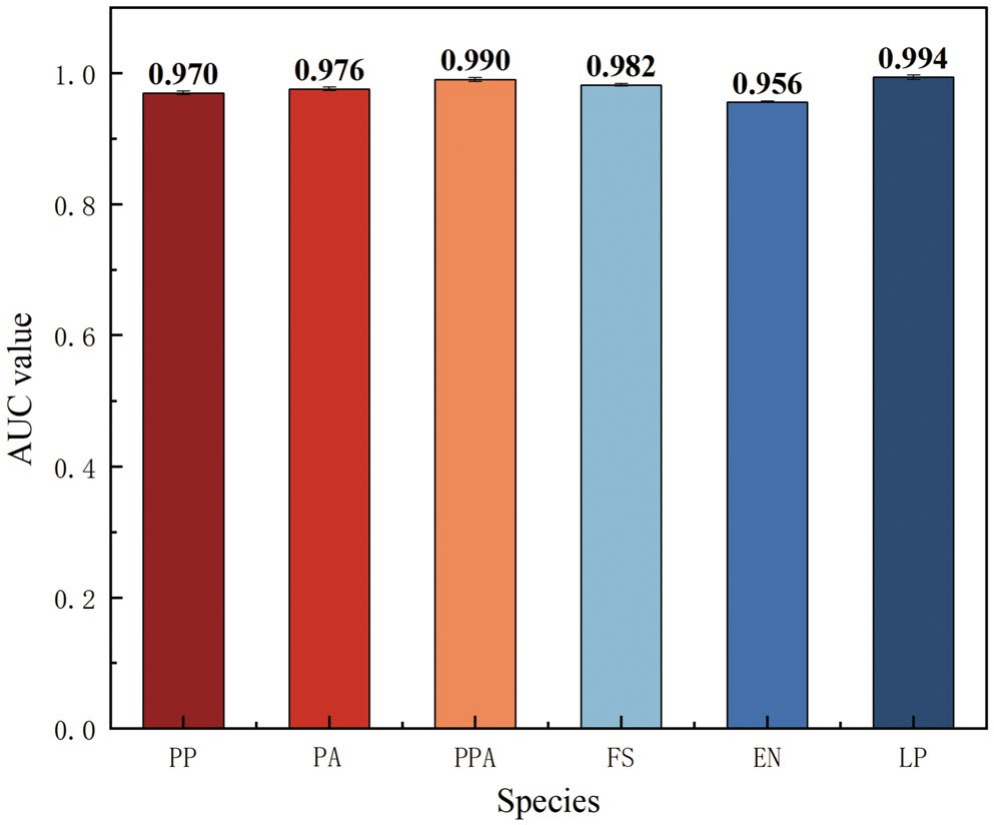
AUC values of typical vegetation restoration species on the QTP.

After ten repetitions, the model obtained the following prediction results: AUC value of *P. pratensis* is 0.970 (TSS = 0.7772), *P. araratica* is 0.976 (TSS = 0.7185), *P. pratensis* var. *anceps* is 0.990 (TSS = 0.7343), *F. sinensis* was 0.982 (TSS = 0.7485), *E. nutans* is 0.956 (TSS = 0.7883), and *L. perenne* is 0.994 (TSS = 0.7735). The AUC values were close to 1, indicating that the models for each species were highly accurate. Additionally, TSS values fell between 0.7185 and 0.7883, corresponding to good to excellent model performance (per TSS evaluation standards: ≤ 0.4 = poor, 0.4–0.7 = moderate, 0.7–0.8 = good, > 0.8 = excellent). Collectively, these dual metrics confirm that all species models were reliable and suitable for subsequent analysis (Figure 3 and Table 2).

**TABLE 2:** AUC and TSS values for each species.

Species	TSS values	AUC values
*P. pratensis*	0.7772 ± 0.0610	0.9702 ± 0.0024
*P. araratica*	0.7185 ± 0.1002	0.9759 ± 0.0026
*P. pratensis* var. *anceps*	0.7343 ± 0.0881	0.9904 ± 0.0030
*F. sinensis*	0.7485 ± 0.1095	0.9820 ± 0.0021
*E. nutans*	0.7883 ± 0.0380	0.9562 ± 0.0011
*L. perenne*	0.7735 ± 0.0909	0.9936 ± 0.0032

### 3.2 Environmental Factors Affecting the Species Distribution of Typical Vegetation Restoration Species on the QTP

The contribution rates of environmental factors and the knife‐edge diagram show that on the QTP, elevation (alt) and annual precipitation (bio12) are the most important environmental factors affecting the six typical vegetation restoration species on the QTP. The sum of the contribution rates of elevation (alt) and annual precipitation (bio12) to the six plants is: *P. pratensis* (80.4%), *P. araratica* (72.8%), *P. pratensis var. anceps* (58.7%), *F. sinensis* (81.7%), *E. nutans* (74.7%), *L. perenne* (46.6%). Next, isothermality (bio3) had a major influence on the distribution of *P. pratensis* , *P. araratica*, *P. pratensis* var. *anceps* and *L. perenne* , while the maximum temperature in the warmest month (bio5) had a significant influence on the distribution of *E. nutans* and the variance of seasonal temperature changes (bio4) had a significant influence on the distribution of *F. sinensis* (Figure 2).

We further analyzed the suitable intervals of the three environmental factors with high contribution rates for the six typical vegetation restoration species on the QTP(Figures S2 and S3). According to the environmental factor response curve, the suitable elevation for *P. pratensis* on the QTP is 2800‐3200 m, the annual average precipitation is 300‐600 mm, and the Isothermality index is 32–33; the suitable elevation for *P. crymophilus* is suitable for elevations of 3500–4100, with an annual precipitation of 450–510 mm and an Isothermality index of 33–35; *P. pratensis* var. *anceps* is suitable for elevations of 2800–3100, and for annual precipitation, suitability increases with increasing annual precipitation in the range of 0‐1000 mm. When the annual precipitation reaches 1000 mm, the suitability does not change much. The suitable range for Isothermality is 32–34. *F. sinensis* is suitable for elevations of 2800–3000 m. When the annual precipitation is 0–500 mm, the suitability gradually increases with the increase of annual precipitation. When the annual precipitation reaches 500 mm, the suitability becomes stable. The suitable range for temperature seasonal change variance is 8000–8100. *E. nutans* is suitable for an elevation of 3100–3300 m, and the suitable range of annual precipitation is 350‐450 mm. The suitability increases with the increase of the maximum temperature of the hottest month. At 0°C–5°C, its suitability remains the same; the suitable elevation range for *L. perenne* is 3000‐3200 m, and its suitability increases with the annual precipitation at 0–500 mm. When the annual precipitation reaches 500 mm, its suitability tends to be stable. Its suitability increases with the increase of isothermality after 20.

### 3.3 Distribution of Contemporary Ecoregions

**FIGURE 4:**
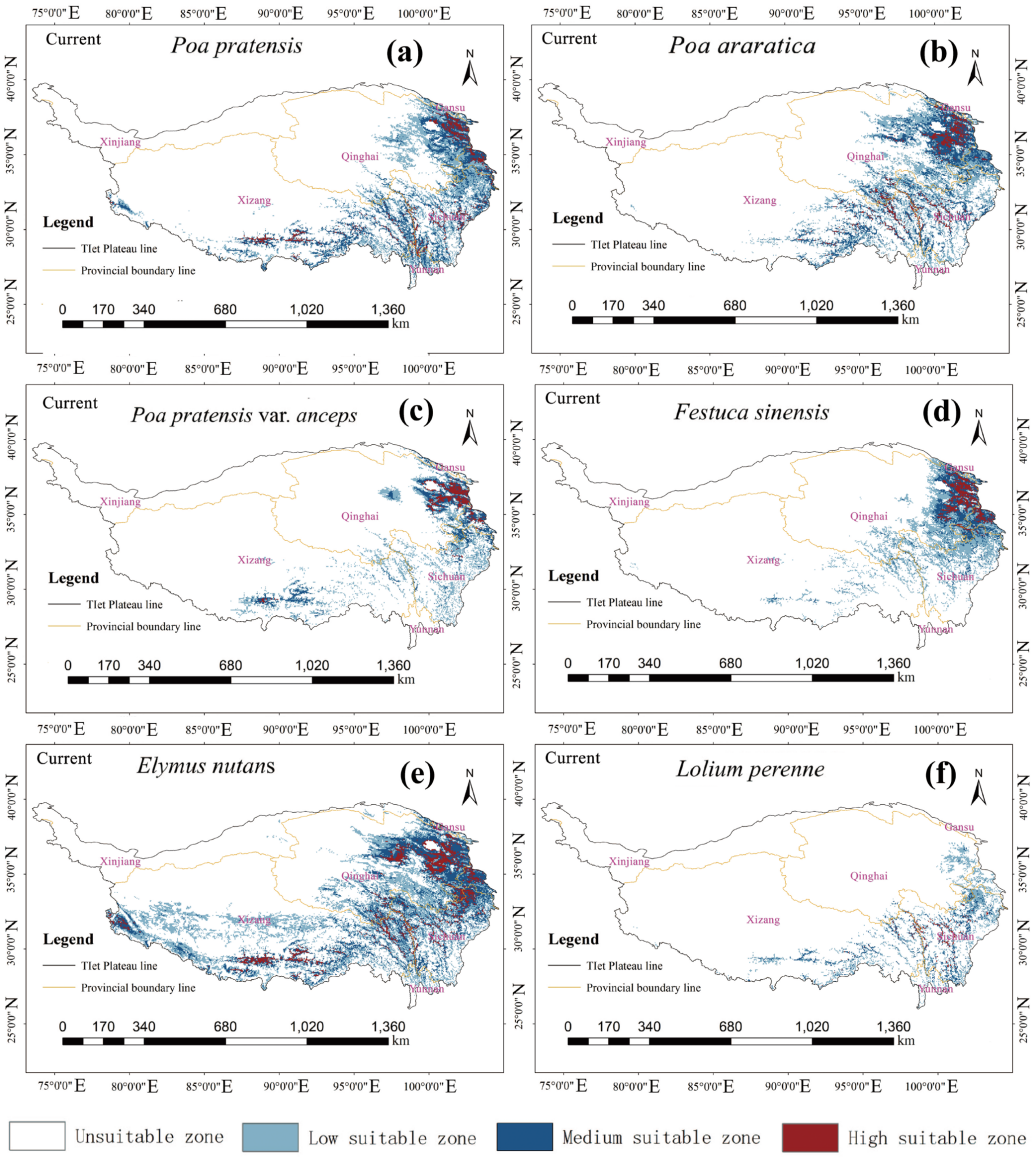
Map of the contemporary distribution of typical vegetation restoration species on the QTP.

In the contemporary climate context, the heavy suitable areas of *P. pratensis* , *P. araratica, P. pratensis* var. *anceps* and *E. nutans* are mainly concentrated in the eastern and northerly parts of the QTP (Figure 4), especially in the eastern part of Qinghai Province and the southwestern part of Gansu Province. In addition, the highly suitable area of *E. nutans* is also distributed in the west and south of the QTP, especially in the southwest and south of the Tibet Autonomous Region (Figure 4), as well as in the north of Yunnan Province and the northwest of Sichuan Province. The heavily suitable area covers an area of up to 8.32 × 10^4^ km^2^. The highly suitable areas of *P. pratensis* and *P. araratica* also extend to the south of the QTP, including the south of the Tibet Autonomous Region, the north of Yunnan Province, and the northwest of Sichuan Province. The southern part of the Qinghai‐Tibet Plateau, including the southern part of the Tibet Autonomous Region, the northern part of Yunnan Province, and the northwestern part of Sichuan Province, where the highly suitable area of *P. pratensis* reaches 3.69 × 10^4^ km^2^, the highly suitable area of *P. araratica* is 3.87 × 10^4^ km^2^ (Table S2), and the heavily suitable area of *P. pratensis* var. The heavy suitable area of *P. pratensis* is distributed in a small area in the southern QTP, with an area of 2.37 × 10^4^ km^2^ (Table S2). The heavy suitable area of *L. perenne* is located in the southeastern QTP, mainly distributed in the northern Sichuan Province and southeastern Tibet. Its heavy suitable area is relatively small, with an area of 0.81 × 10^4^ km^2^. *E. nutans* has the most widespread distribution, with a total suitable area of about 61% of the total area of the QTP, or 80.73 × 10^4^ km^2^ (Table S2).

### 3.4 Prediction of the Future Suitable Habitat of Typical Vegetation Restoration Species on the QTP

We used MaxEnt to analyze the distribution of several typical species of vegetation restoration on the QTP under different climate scenarios (RCP2.6, RCP6.0, RCP8.5) in the future (2050s, 2070s), and predict the changes in the distribution of each species over time (Figures S4 and 5).

**FIGURE 5:**
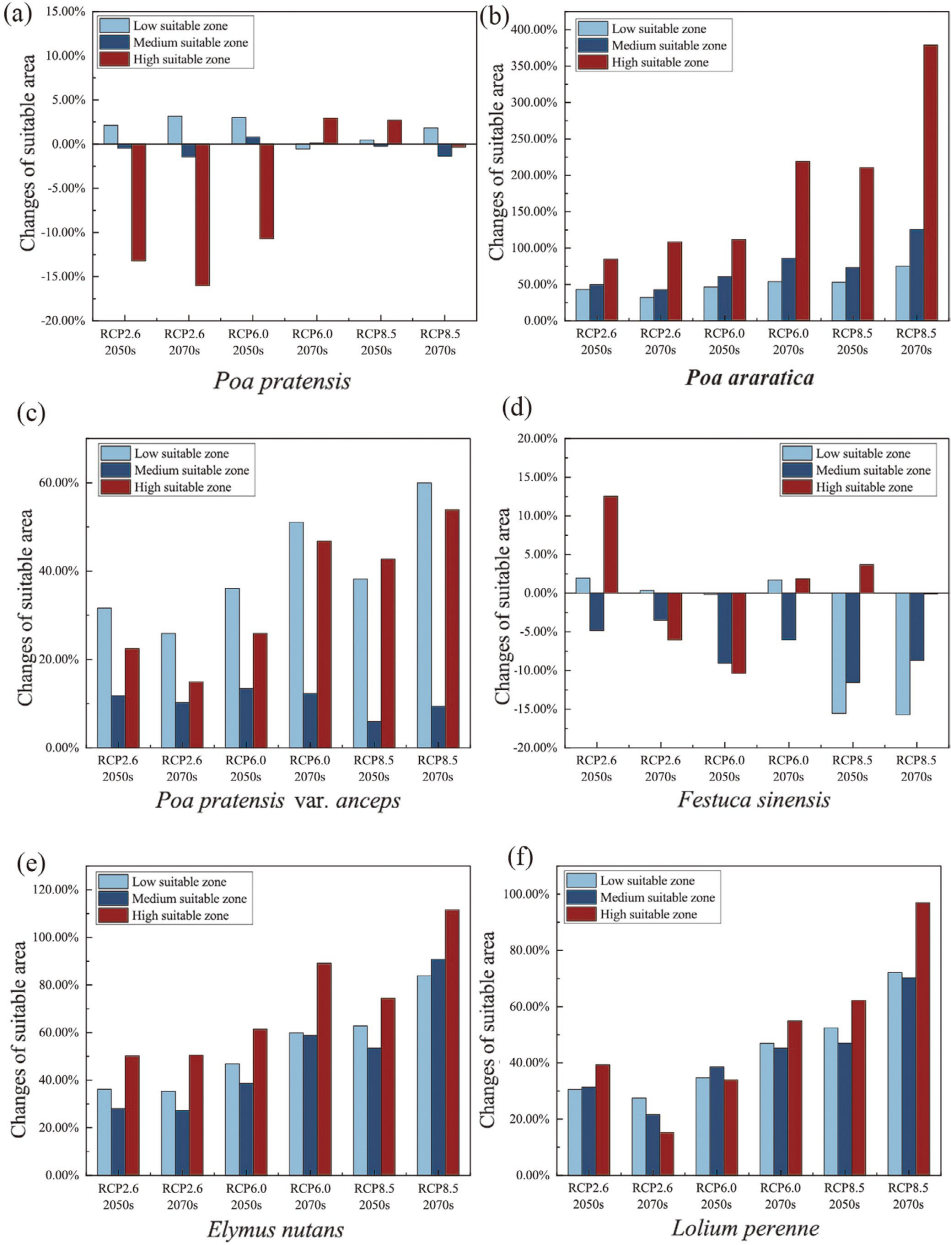
Area changes of each plant at different times under different climate scenarios.

Compared with the contemporary predicted suitable distribution area, the low, medium, and high suitable zones of *P. araratica*, *P. pratensis* var. *anceps*, *E. nutans* , and *L. perenne* have all expanded under the background of global warming (Figure 5). Among these vegetation restoration species, the largest increase in suitable zone is *P. araratica*, of which the range of increase in highly suitable zone is 14.98%–378.88%; the range of increase in moderately suitable area ranged from 49.85% to 125.58%. Under the RCP8.5 climate scenario, the area of moderate and heavy suitable habitats in the 2070s increased the most; the next species with a high growth range was *E. nutans* . Under the RCP8.5 climate scenario, the growth range was the largest in the 2070s, with a 90.79% increase in moderate suitable habitats and 111.66% in highly suitable habitats (Figure 5).

However, it is worth noting that the area of *P. pratensis* in the moderately suitable and highly suitable zones under the RCP2.6 scenario decreases over time, with the highest decrease in the 2070s, a decrease of 1.44% in the moderately suitable zone and 16.02% in the highly suitable zone. Under the RCP6.0 climate scenario, the area of moderately suitable area increased slightly, the area of the heavy suitable zone decreased in the 2050s by 10.71%, but increased in the 2070s by 2.95%. Under the RCP8.5 climate scenario, the area of the heavy suitable zone for *P. pratensis* overall decreased, but the decrease was small. Under different climate scenarios, the area of moderate suitability for *F. sinensis* decreased by 3.48%–11.56%. Under the RCP8.5 climate scenario, the area of moderate suitability decreased the most, while the area of heavy suitability decreased or increased at different times under different climate scenarios, with the RCP2.6 climate scenario showing the highest growth rate of 12.53% in the severe suitability zone, and the RCP6.0 climate scenario showing the largest decrease of 10.37% in the severe suitability zone in the 2050s (Figure 5).

### 3.5 Future Migratory Trends of Species for the Restoration of Vegetation on the QTP

**FIGURE 6:**
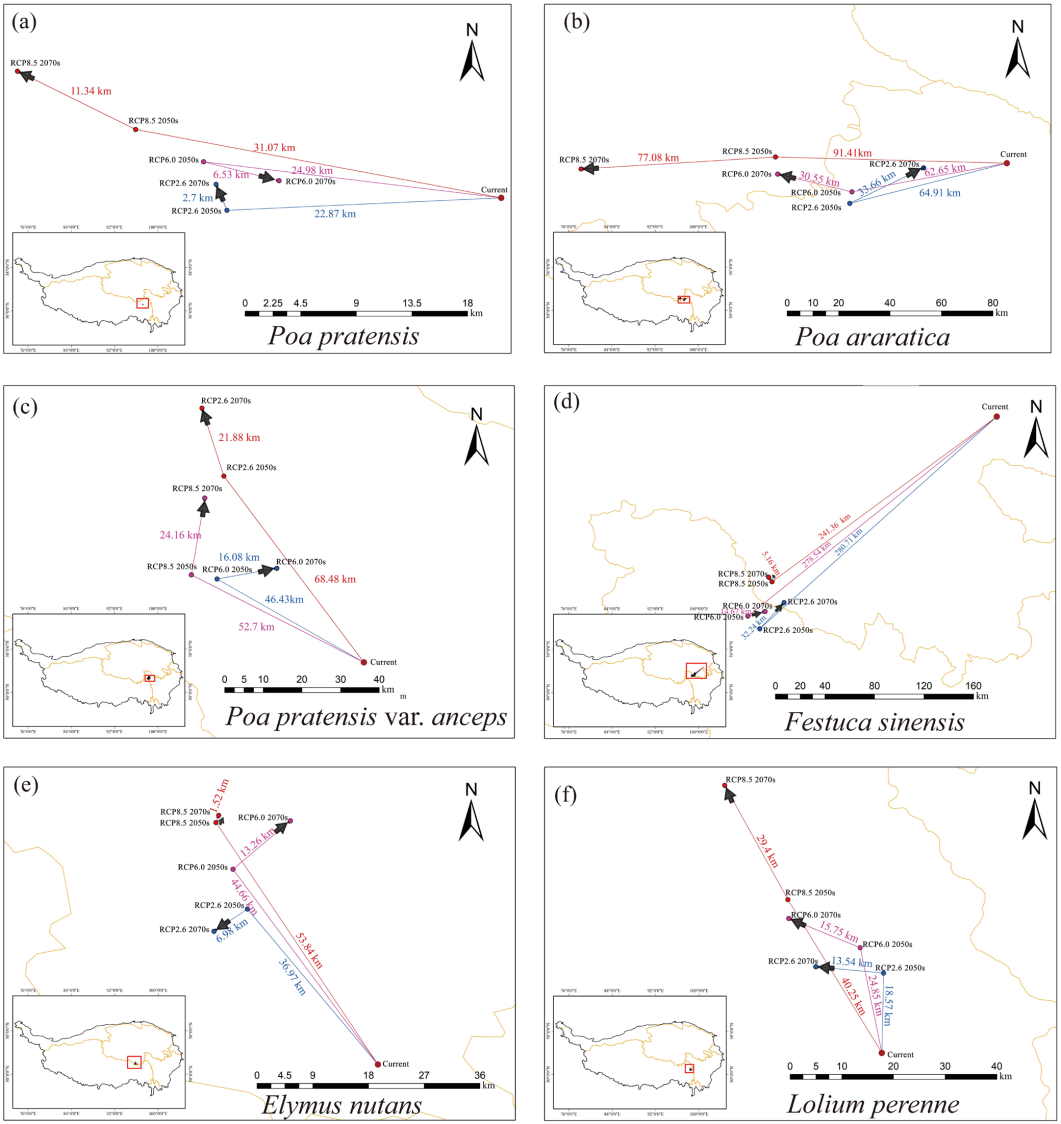
Migration map of the center of mass of typical vegetation restoration species on the QTP.

ArcGIS was used to calculate the centroid position and migration direction of vegetation restoration species. The results showed that under the global warming climate scenario, *P. pratensis* , *P. pratensis* var. *anceps*, *E. nutans* , and *L. perenne* migrated to the northwest (Figure 6a,c,e,f), *P. araratica* had a tendency to migrate westward (Figure 6b), and *F. sinensis* had a tendency to migrate southwestward (Figure 6d). Under the RCP8.5 climate scenario, the migration distance of the six species was the greatest. The contemporary centroid of *P. pratensis* is located in Changdu City, Tibet Autonomous Region, at coordinates 97.444° E, 31.146° N. From the 2050s to the 2070s, it is projected to migrate in a northwest direction, ultimately reaching coordinates 97.021° E, 31.256° N (Figure 6a and Table S3), with a total migration distance of 42.42 km under the RCP8.5 scenario. The contemporary centroid of *P. pratensis* var. *anceps* is located in Ganzi Tibetan Autonomous Prefecture, Sichuan Province, at coordinates 98.676° E, 32.422° N. From the 2050s to the 2070s, it is projected to migrate in a northwest direction, reaching coordinates 98.223° E, 33.132° N (Figure 6c and Table S3), with a total migration distance of 90.36 km under the RCP8.5 scenario (Table S4). The current centroid of *E. nutans* is located in Changdu City, Tibet Autonomous Region, at coordinates 95.986° E, 31.809° N. In the 2050s, it is projected to migrate in a northwest direction to coordinates 95.704° E, 32.230° N, followed by a slight northeast shift in the 2070s to coordinates 95.709° E, 32.243° (Figure 6e and Table S3), with a total migration distance of 55.36 km under the RCP8.5 scenario (Table S4). The current centroid of *L. perenne* is located in Changdu City, Tibet Autonomous Region, at coordinates 98.589° E, 30.671° N. From the 2050s to the 2070s, it is projected to migrate in a northwest direction, ultimately reaching coordinates 98.260° E, 31.230° N (Figure 6f and Table S3), with a total migration distance of 69.65 km under the RCP8.5 scenario (Table S4). The current centroid of *P. araratica* is located in Changdu City, Tibet Autonomous Region, at coordinates 98.028° E, 32.187° N. By the 2070s, it is projected to migrate to coordinates 96.240° E, 32.163° N (Figure 6b and Table S3), with a total migration distance of 168.49 km under the RCP8.5 scenario (Table S4). The current centroid of *F. sinensis* is located in Tongde County, Hainan Tibetan Autonomous Prefecture, Qinghai Province, at coordinates 100.933° E, 34.751° N. Under the RCP8.5 climate scenario, by the 2070s, it is projected to migrate in a southwest direction to coordinates 98.942° E, 33.351° N (Figure 6d and Table S3), with a total migration distance of 246.51 km (Table S4).

### 3.6 Niche Breadth and Niche Overlap

**FIGURE 7:**
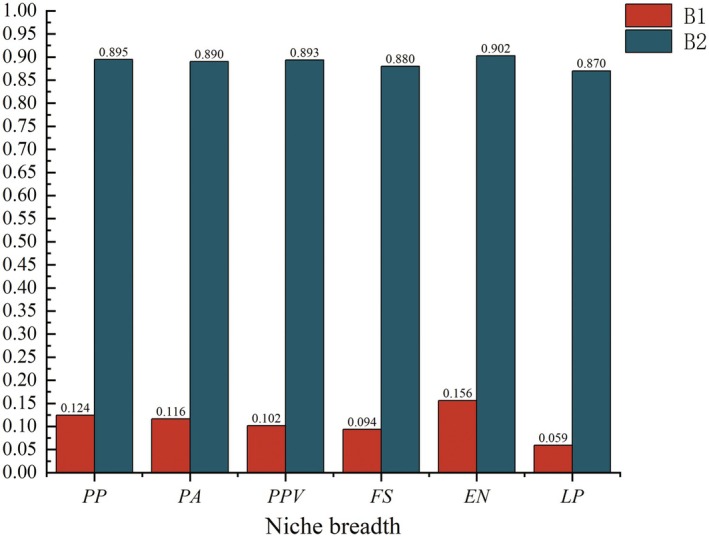
Niche breadth for typical vegetation restoration species on the QTP.

The niche breadth of six typical vegetation restoration species on the QTP was calculated using ENMTools (Figure 7). The results showed that the niche breadth of *E. nutans* was the highest (*B*1 = 0.156, *B*2 = 0.902), followed by *P. araratica* (*B*1 = 0.124, *B*2 = 0.895) and *P. pratensis* var. *anceps* (*B*1 = 0.102, *B*2 = 0.893).

Using the ENMTools tool to predict the niche overlap index of six species (Table 3). The highest overlap index was found between *P. araratica* and *P. pratensis* (*D* = 0.75/*I* = 0.94). Following that, both *P. araratica* and *P. pratensis* showed relatively high ecological overlap with *F. sinensis* and *E. nutans* (*D* values exceeding 0.74, *I* values exceeding 0.90). The ecological niche overlap of *L. perenne* with other species is relatively low, especially with *E. nutans* and *P. pratensis* var. *anceps* (*D* = 0.48, *I* = 0.77).

**TABLE 3:** Niche overlap of the six plants.

D (above the diagonal)/I (below the diagonal)	EN	FS	LP	PA	PP	PPV
EN	1.00	0.65	0.48	0.74	0.74	0.61
FS	0.88	1.00	0.52	0.74	0.68	0.54
LP	0.77	0.81	1.00	0.56	0.61	0.48
PC	0.93	0.94	0.83	1.00	0.75	0.55
PP	0.94	0.91	0.86	0.94	1.00	0.59
PPV	0.83	0.80	0.77	0.81	0.82	1.00

*Note:* Color shades indicate the diagonal cells (value 1.00) that represent the niche overlap of a species with itself (value = 1, complete overlap). The shading has no other statistical meaning.

## 4 Discussion

### 4.1 Environmental Factors Affecting the Species Distribution of Typical Vegetation Restoration on the QTP

This study conducted an in‐depth analysis of the distribution of suitable zones and environmental factors for six typical vegetation restoration species on the QTP based on the MaxEnt model. The results show that elevation (alt) and annual precipitation (bio12) are the two environmental factors that have a significant impact on the distribution of the six plant species. Therefore, elevation (alt) and annual precipitation (bio12) are the primary factors influencing the distribution of these species. This indicates that in the unique cold alpine ecosystem of the QTP, elevation and precipitation have a decisive influence on the distribution of plants growing in the plateau (Chu et al. 2024; Shi et al. 2023). The dominant role of elevation is physiologically underpinned by its integrated control over multiple abiotic stressors: at higher elevations, plants face hypobaric hypoxia, intensified ultraviolet (UV) radiation, lower ambient temperatures, and shortened growing seasons. These conditions directly regulate plant physiological processes such as photosynthetic efficiency, stomatal conductance, and resource allocation strategies, leading to adaptive shifts in leaf morphology and root architecture (Körner 2007; Shi et al. 2015). Consequently, elevation acts as a “master variable” that varies with multiple limiting resources, explaining its high contribution in niche models. Annual precipitation (bio12) directly regulates water availability, influencing stomatal regulation and turgor maintenance. This aligns with QTP restoration goals, where water availability is a key constraint.

Besides elevation and annual precipitation, isothermality (bio3) is an important environmental factor influencing the distribution of *P. pratensis* , *P. araratica*, *P. pratensis* var. *anceps*, and *L. perenne* . This may be because isothermality (bio3) reflects the stability of the diurnal temperature range, which is crucial for maintaining cellular homeostasis (Mosco 2019; Peng et al. 2025). The maximum temperature of the hottest month (bio5) also affects the distribution of *E. nutans* , while temperature seasonality (bio4) is a significant environmental factor for *F. sinensis* . Temperature seasonality (bio4) affects *F. sinensis* by imposing predictable seasonal cues; high seasonality requires precise phenological synchronization (e.g., flowering, dormancy) to avoid frost damage, which may drive adaptive divergence in thermal reaction norms (Chen et al. 2022). This indicates that temperature variation has a considerable impact on the distribution of these species (Zou et al. 2020).

The research results indicate that the suitable elevation (alt) for *P. pratensis* , *P. araratica*, *P. pratensis* var. *anceps*, *F. sinensis* , *E. nutans* , and *L. perenne* is relatively high, and the most suitable annual precipitation (bio12) is around 500 mm. This aligns with the conditions often associated with species used for vegetation restoration in the QTP (Sun et al. 2022). The suitability of *P. pratensis* var. *anceps* and *L. perenne* to annual precipitation (bio 12) increases as annual precipitation increases, and they still have good adaptability when annual precipitation (bio 12) is high. Therefore, they have a strong adaptability to water conditions, which is consistent with the results of studies on the impact of water on this plant (Chastain et al. 2015; Wei et al. 2021). The suitability of *L. perenne* increases with the increase of isothermality (bio3), indicating that *L. perenne* is more adaptable to places with higher climate stability, such as Sichuan and Yunnan provinces(Barták et al. 1998). The suitable range for isothermality (bio3) of *P. pratensis* var. *anceps* is 32–34, making it more adaptable to areas with lower climate stability. Therefore, in the QTP at higher elevations, where climate stability is lower and precipitation is higher, *P. pratensis* var. *anceps* can be preferentially selected (Dong et al. 2020). The suitability of *E. nutans* for the maximum temperature of the hottest month remains constant between 0°C–5°C, and beyond 5°C, its suitability increases with rising temperatures. This indicates that this can cope with the temperature increases caused by climate warming, which is consistent with previous research findings regarding the effects of rising temperatures on *E. nutans* (Ren et al. 2010).

### 4.2 Changes in Typical Vegetation Restoration Species on the QTP in the Context of Current and Future Climate Warming

We used the MaxEnt model to predict the current suitable distribution of typical vegetation restoration species in the QTP. The results show that the heavily suitable zones for *P. pratensis* , *P. araratica*, *P. pratensis* var. *anceps*, *F. sinensis* , and *E. nutans* are mainly concentrated in the northern eastern region of the QTP, particularly in the eastern part of Qinghai Province and the southwestern part of Gansu Province. *E. nutans* has the most extensive suitable distribution, it shows that it has a strong adaptability (Li et al. 2021; Sun et al. 2022), and can be widely applied in vegetation restoration across various regions of the QTP. The highly suitable zones for *P. pratensis* and *P. araratica* also extend to the southern part of the QTP, while there is a small distribution of heavily suitable zones for *P. pratensis* var. *anceps* in the southern QTP. The high suitable zone of *L. perenne* is mainly located in the southeast part of the QTP, which is consistent with the fact that *L. perenne* is more suitable for areas with higher climate stability (Berone et al. 2007).

In the context of global warming, this study projected changes in the suitable zones of various vegetation restoration species under different climate scenarios. The results indicate that the low, medium, and high suitable zones for *P. araratica*, *P. pratensis* var. *anceps*, *E. nutans* , and *L. perenne* have all expanded. In particular, *P. araratica* and *E. nutans* show significant increases in their suitable zone areas. Under the RCP8.5 climate scenario, the range of increase for the highly suitable zone of *P. araratica* is 14.98%–378.88%, while the medium suitable zone area shows an increase of 49.85%–125.58%. In the 2070s, the medium suitable zone for *E. nutans* increased by 90.79%, and the highly suitable zone increased by 111.66%. These results indicate that with climate warming, the growth potential of these species will further enhance. The suitable zone areas for *P. pratensis* and *F. sinensis* show an overall trend of contraction under the context of climate warming, but the extent of this contraction is relatively small, indicating that they are not severely affected by climate warming and demonstrate a certain degree of adaptability to these changes (Hua et al. 2019). In the context of global warming, these six species can continue to serve as typical vegetation restoration species in the QTP.

Under the climate scenario of global warming, species such as *P. pratensis* , *P. pratensis* var. *anceps*, *E. nutans* , and *L. perenne* primarily migrate northwest, while *P. araratica* migrates westward and *F. sinensis* migrates southwest, exhibiting a trend of migration from lower to higher altitudes. This may be due to the rise in temperatures and changes in precipitation patterns caused by global climate warming, prompting these species to move toward “colder” regions to adapt to environmental changes. Some studies have also indicated that the distribution patterns of most flora and fauna show a significant trend of migrating from low‐altitude areas to high‐altitude areas (Parmesan and Yohe 2003; Root et al. 2003; Thuiller 2003; Wilson et al. 2005). These migration trends are closely related to the changes in temperature and precipitation patterns induced by climate change and reflect the ability of species to make adjustments in response to environmental changes during the process of climate change (Liu et al. 2018; Umair et al. 2023; Wen et al. 2022; Yao et al. 2025; Zhao et al. 2024).

### 4.3 Discussion on Niche Breadth and Niche Overlap

We used the ENMTools tool to calculate the niche breadth and the ecological niche overlap index (Figure 7 and Table 3). The results show that *E. nutans* has the highest niche breadth (*B*1 = 0.156, *B*2 = 0.902), indicating its strong adaptability to the environment, which is consistent with its extensive suitable habitat area, potentially due to its ability to adapt not only to high altitudes but also to temperatures effectively than other species, allowing it to thrive under various environmental conditions in the QTP (Li et al. 2024; Ren et al. 2010; Shi et al. 2010). In addition, *P. pratensis* and *F. sinensis* also have high niche widths (B1 values of 0.124 and 0.102, respectively with B2 values exceeding 0.89), indicating they also possess good environmental adaptability. The results of the niche overlap index show the overlap between *P. pratensis L. perenne* is the highest (*D* = 0.75, *I* = 0.94), a high ecological similarity between them. Furthermore, *P. pratensis* and *L. perenne* exhibit high overlap with *F. sinensis* and *E. nutans* , respectively (*D* values 0.74, *I* values all exceeding 0.90), indicating that their niches are highly overlapping. If planted in the same resource‐limited area, there may be a potential risk of competition that requires field validation to confirm if growth and distribution would be restricted (An et al. 2023; Li et al. 2014). However, species with high niche overlap have similar resource requirements, and similar niche widths indicate comparable adaptability to the environment. When conducting vegetation restoration, providing certain nutrient resources and selecting species with similar niche widths for co‐planting may help mitigate potential competition (Gilbert 2012; Price et al. 2014). *L. perenne* has relatively low niche overlap with other species, particularly with *E. nutans* and *P. pratensis* var. *anceps* (*D* = 0.48, *I* = 0.77). But analyzing the distribution of *L. perenne* 's suitable habitat and environmental factors compared to other species, *L. perenne* has significant differences in its suitable habitat and environmental factors compared to other species. We recommend avoiding co‐planting *L. perenne* with other species in resource‐limited areas, as their distinct environmental requirements may lead to mismatched growth dynamics. Therefore, when selecting species for co‐planting in vegetation restoration, it is essential to consider the niche overlap between species, their adaptability, and the environmental conditions. When the niche overlap is high, it is advisable to avoid combinations of species with significantly different competitive abilities, such as *E. nutans* with other species, as the high adaptability of *E. nutans* may create a potential risk of competition that requires field test (Odriozola et al. 2017).

### 4.4 Limitations and Prospects

While our MaxEnt‐based predictions provide valuable insights for vegetation restoration planning, it is important to acknowledge certain limitations of the modeling framework employed. Specifically, our use of a simple random 75/25 split for model training and validation may introduce spatial autocorrelation bias, as occurrence points in close geographic proximity are often not independent. This can lead to over‐optimistic model performance estimates; the initial data partitioning does not fully account for spatial structure in the data, and adopting spatial block (or checkerboard) cross‐validation would provide more robust estimates of model transferability and performance under novel environmental conditions (Valavi et al. 2019).

While MaxEnt performs well in predicting the impacts of climate change on species distributions, its adaptability to extreme climates still has certain limitations (Kumar 2012; Warren et al. 2021). The model relies on current species distribution data and input environmental variables, making it difficult to capture unprecedented climate extremes (e.g., heatwaves, droughts) (Ren et al. 2025). Specifically, MaxEnt assumes that species‐environment relationships are stationary and transferable to future conditions; however, extreme events often push species beyond their physiological thresholds, leading to non‐linear responses or rapid mortality that the model cannot reliably predict based on historical correlations alone. Furthermore, the model does not inherently account for phenotypic plasticity or rapid evolutionary adaptation, which may buffer or exacerbate the impacts of extreme stressors in real‐world ecosystems. Therefore, future research should further increase the study and discussion of extreme climates, potentially by integrating process‐based models that simulate physiological mechanisms or by incorporating extreme‐event scenarios into species distribution projections.

## 5 Conclusions

Under global warming, this study evaluated the restoration potential of six herbaceous species on the Qinghai‐Tibet Plateau (QTP) by integrating habitat suitability modeling, environmental factor analysis, niche characteristics, and range shift trends. Key findings revealed that *P. pratensis* , *P. araratica*, and *F. sinensis* —species with high niche overlap (*D* > 0.74) and shared adaptability to moderate elevation and high precipitation—are prioritized for northeastern QTP (eastern Qinghai, southern Gansu, and northern Yunnan), though their mixed sowing requires competition management. The widespread *E. nutans* , exhibiting the broadest niche breadth (*B*1 = 0.156) and climate resilience, emerged as a pioneer species for cross‐regional restoration, yet its planting should exclude ecologically overlapping species to avoid the potential risk of competitive exclusion, which requires field validation. *P. pratensis* var. *anceps*, tolerant to precipitation increases, suits northeastern climate‐vulnerable zones, while *Lolium perenne* is restricted to climatically stable areas in Sichuan and northern Yunnan. Critically, all species showed westward range shifts toward higher elevations indicating that the pool of available restoration species for high‐altitude areas in western QTP will expand. By applying the MaxEnt model to predict typical vegetation restoration species on the Qinghai‐Tibet Plateau, this study provides a reasonable basis for species selection and combination in QTP vegetation restoration projects under future climate warming scenarios.

Future research should focus on three directions to enhance the rigor and applicability of restoration strategies. First, replace random data splits with spatially explicit cross‐validation to reduce bias, and refine nonlinear responses using CMIP6 multi‐model scenarios. Second, conduct in situ experiments to verify species' responses to environmental stresses. Third, carry out corresponding field vegetation restoration experiments in different regions, validate and monitor the results over the long term, and form replicable and referenceable samples for the selection and combination of restoration species.

## Author Contributions

**Ying Yang:** formal analysis (equal), investigation (equal), methodology (equal), visualization (equal), writing – original draft (equal). **Chen‐Di Wang:** funding acquisition (equal), investigation (equal). **Yan‐Gang Yin:** funding acquisition (equal), investigation (equal). **Dong Han:** funding acquisition (equal), investigation (equal). **Yue Zhong:** funding acquisition (equal), investigation (equal). **Bo Wang:** conceptualization (equal), methodology (equal). **Guo‐Ying Zhou:** conceptualization (equal), funding acquisition (equal), resources (equal), supervision (equal), writing – review and editing (equal).

## Funding

This study was supported by the Qinghai Natural Science Foundation Team Program (2023‐ZJ‐902T).

## Conflicts of Interest

The authors declare no conflicts of interest.

## Supporting information


**Figure S1:** Photographs of six typical vegetation restoration species on the QTP. Reprinted from Plant Photo Bank of China (https://ppbc.iplant.cn/), with permission from the copyright holder.
**Figure S2:** Response curves of environmental factors affecting the distribution of typical species for vegetation restoration on the QTP.
**Figure S3:** Ecological factor training gain based on MaxEnt model prediction results.
**Figure S4:** Distribution of potential suitable zones for each plant under different climate scenarios (RCP2.6 RCP6.0 RCP8.5) for different time periods (2050s, 2070s).
**Table S1:** The 28 environmental factors used by the MaxEnt model.
**Table S2:** Predict the suitable area of *Sinolimprichtia* plants in different periods(×10^4^ km^2^).
**Table S3:** Centroid coordinates of typical vegetation restoration species on the QTP.
**Table S4:** Centroid migration distances of species under different climate scenarios.

## Data Availability

Climate data can be obtained from Worldclim (http://www.worldclim.org), Soil factor data were obtained from the National Tibetan Plateau Data Center (https://data.tpdc.ac.cn/). Data distribution points of six typical vegetation restoration species and the QTP boundary mask file are openly available in Figshare (https://doi.org/10.6084/m9.figshare.31109302.v1, DOI: 10.6084/m9.figshare.31109302).
