# Supplementary material for: Predict Suitable Restoration Areas for Typical Vegetation Restoration Species on the Qinghai‐Tibetan Plateau Based on MaxEnt
Source: Ecol Evol. 2026 Jun 19;16(6):e73857. doi: 10.1002/ece3.73857 (PMC13282462; doi:10.1002/ece3.73857)
Supplement: Supplementary file 1 — Figure S1: Photographs of six typical vegetation restoration species on the QTP. Reprinted from Plant Photo Bank of China (https://ppbc.iplant.cn/), with permission from the copyright holder. Figure S2: Response curves of environmental factors affecting the distribution of typical species for vegetation restoration on the QTP. Figure S3: Ecological factor training gain based on MaxEnt model prediction results. Figure S4: Distribution of potential suitable zones for each plant under different climate scenarios (RCP2.6 RCP6.0 RCP8.5) for different time periods (2050s, 2070s). Table S1: The 28 environmental factors used by the MaxEnt model. Table S2: Predict the suitable area of Sinolimprichtia plants in different periods(×104 km2). Table S3: Centroid coordinates of typical vegetation restoration species on the QTP. Table S4: Centroid migration distances of species under different climate scenarios. [file ECE3-16-e73857-s001.docx]

**Supplementary Material**

Figure S1: Photographs of six typical vegetation restoration species on the QTP.Reprinted from Plant Photo Bank of China (https://ppbc.iplant.cn/), with permission from the copyright holder.


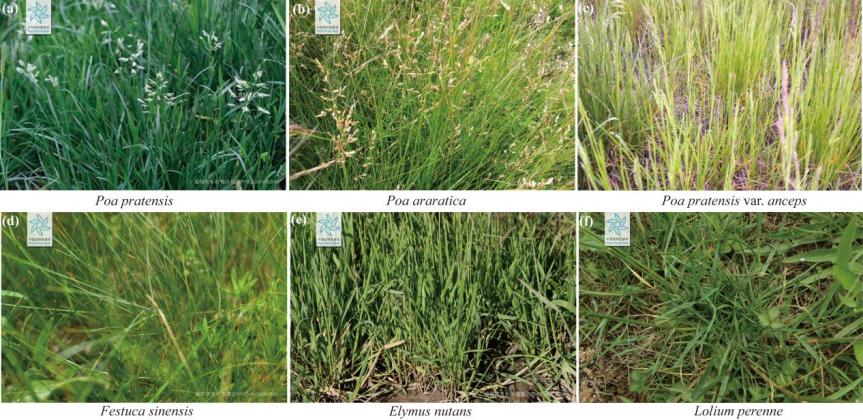


**Figure S2:** Response curves of environmental factors affecting the distribution of typical species for vegetation restoration on the QTP.


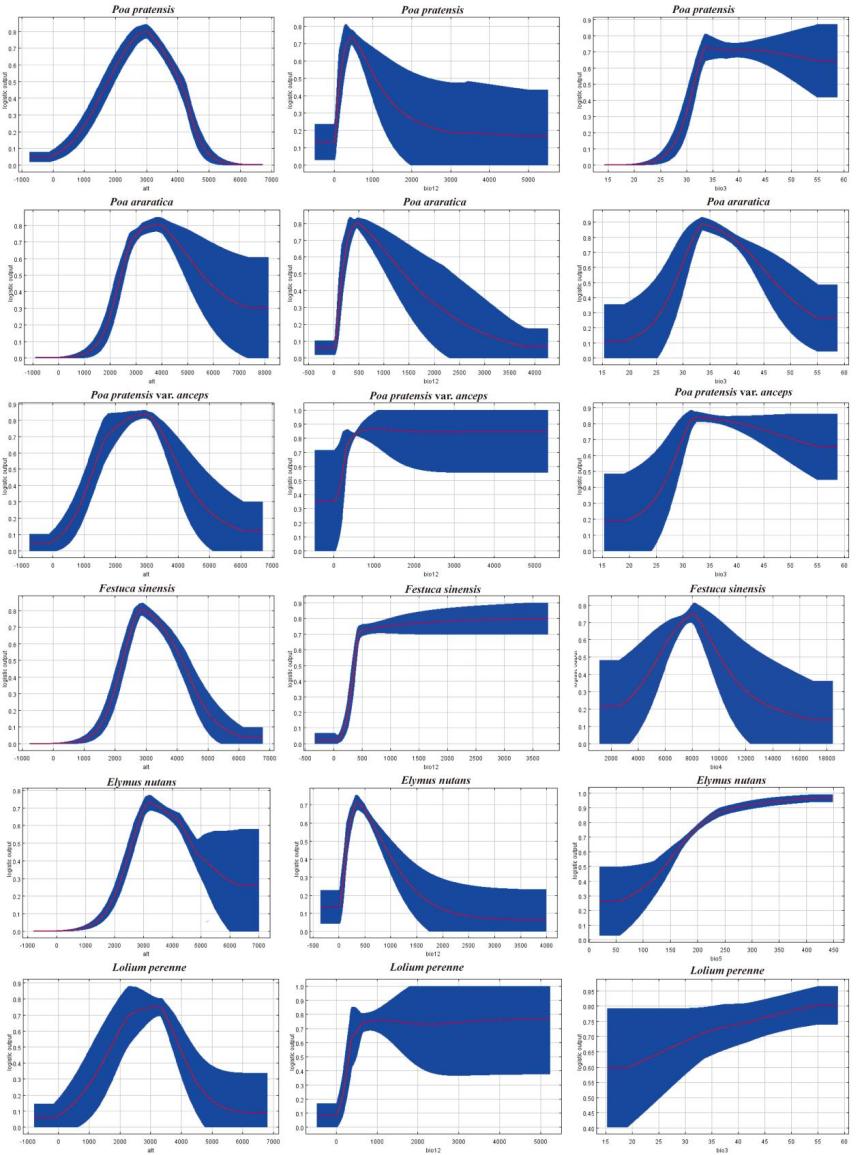


**Figure S3**: Ecological factor training gain based on MaxEnt model prediction results.


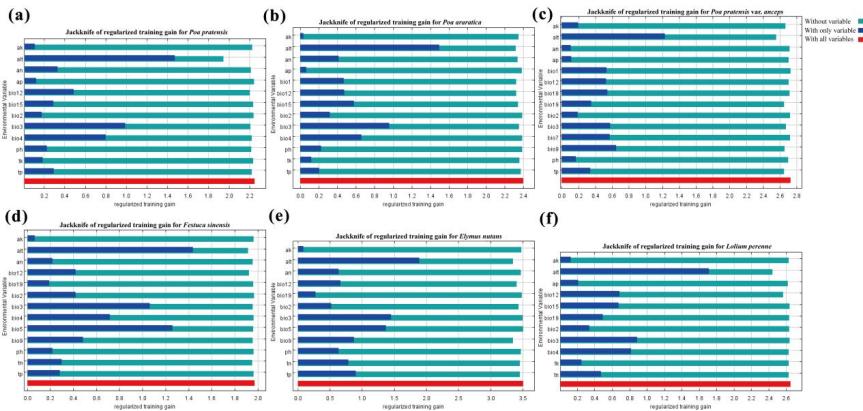


**Figure S4:** Distribution of potential suitable zones for each plant under different climate scenarios (RCP2.6 RCP6.0 RCP8.5) for different time periods (2050s 2070s).


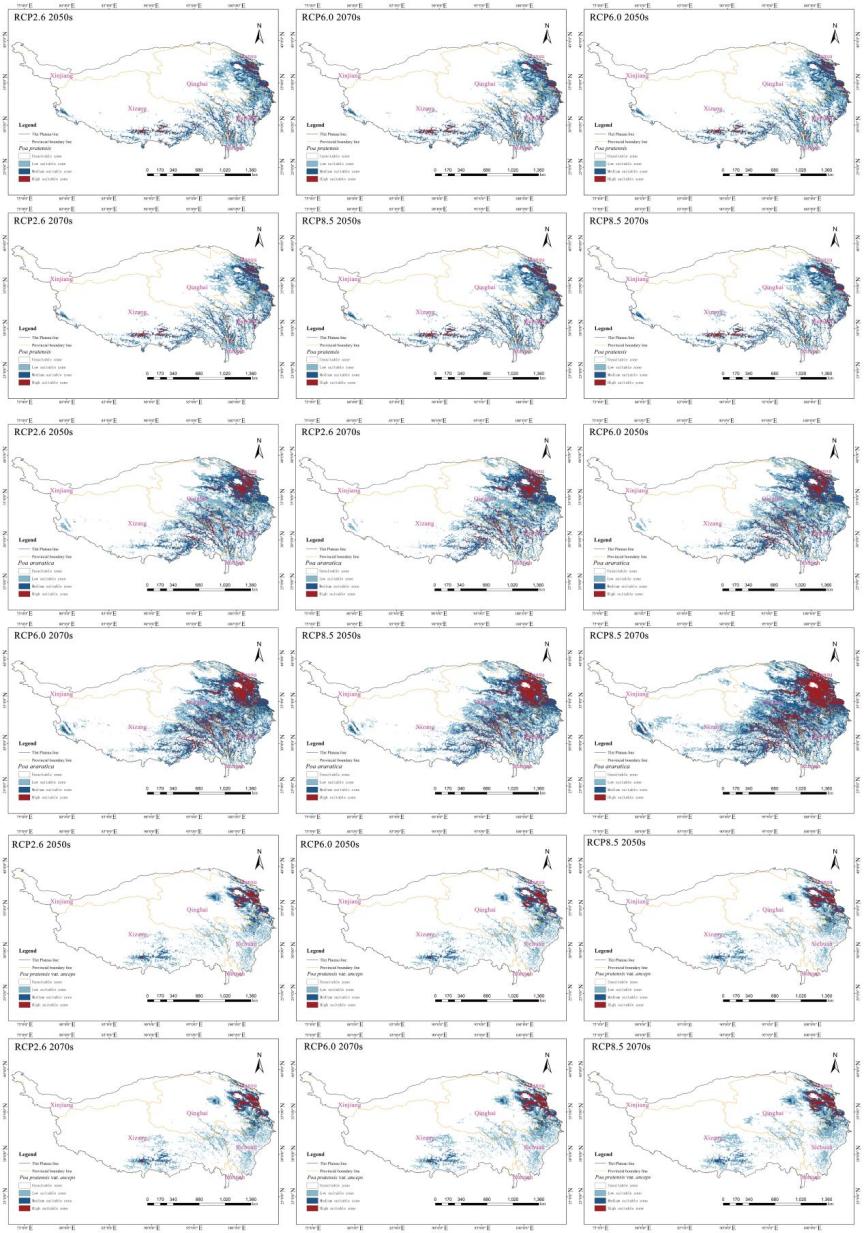


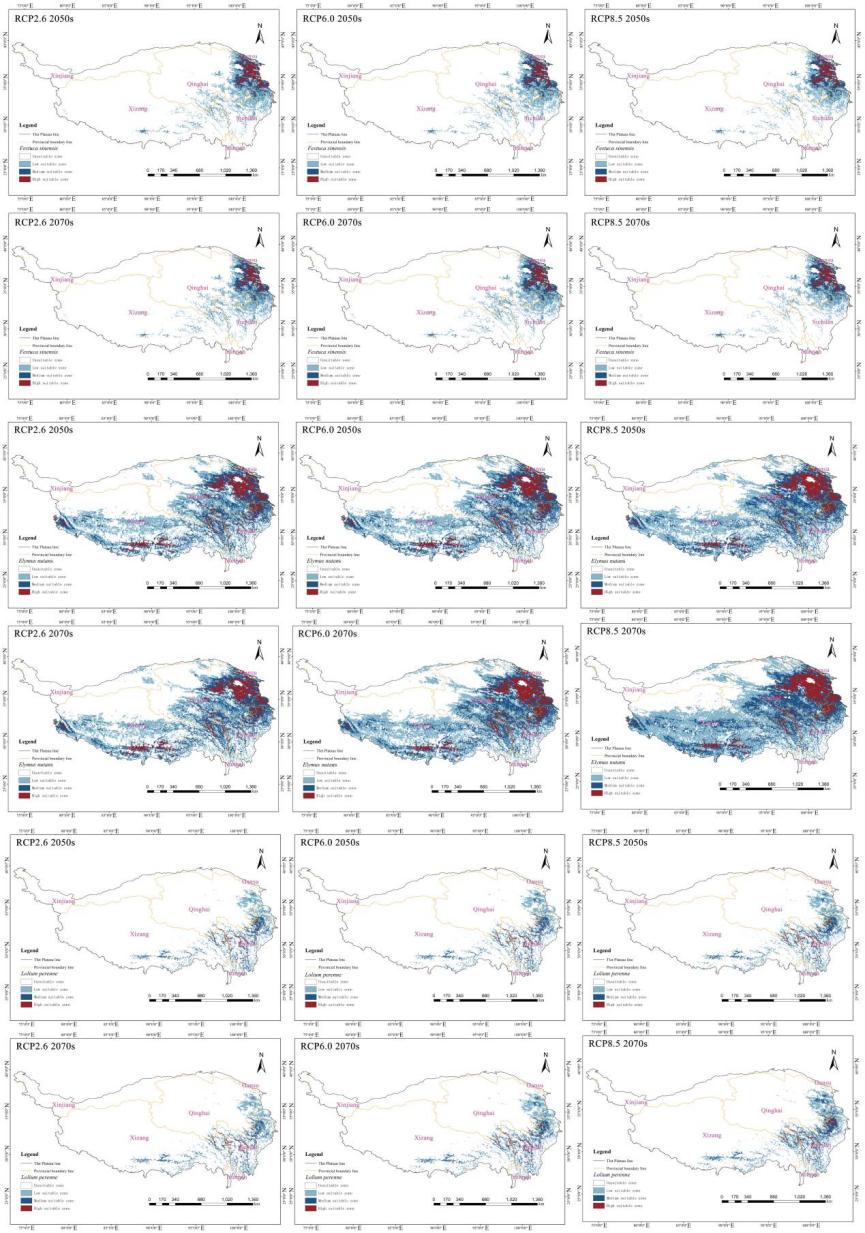


**Supplementary Material**

**Table S1 :** The 28 environmental factors used by the MaxEnt model.

| Variable | Description |
| --- | --- |
| Bio1 | Annual mean temperature（℃） |
| Bio2 | Mean diurnal range（℃） |
| Bio3 | Isothermality（%） |
| Bio4 | Temperature seasonality (standard deviation × 100) (℃) |
| Bio5 | Max temperature of warmest month（℃×10） |
| Bio6 | Min temperature of coldest month（℃） |
| Bio7 | Temperature annual range（℃） |
| Bio8 | Mean temperature of wettest quarter（℃） |
| Bio9 | Mean temperature of driest quarter（℃） |
| Bio10 | Mean temperature of warmest quarter（℃） |
| Bio11 | Mean temperature of coldest quarter（℃） |
| Bio12 | Annual precipitation（mm） |
| Bio13 | Precipitation of wettest month（mm） |
| Bio14 | Precipitation of driest month（mm） |
| Bio15 | Precipitation seasonality（%） |
| Bio16 | Precipitation of wettest quarter（mm） |
| Bio17 | Precipitation of driest quarter（mm） |
| Bio18 | Precipitation of warmest quarter（mm） |
| Bio19 | Precipitation of coldest quarter（mm） |
| Alt | Altitude（m） |
| AK | Available potassium（mg/kg） |
| AN | Available nitrogen（mg/kg） |
| AP | Available phosphorus（mg/kg） |
| TK | Total potassium（g/kg） |
| TN | Soil total nitrogen（g/kg） |
| TP | Total phosphorus（g/kg） |
| PH | Potential hydrogen |
| SOM | Soil organic matter（g/kg） |

**Table S2:** Predict the suitable area of *Sinolimprichtia* plants in different periods(×10^4^ km^2^).

| Species | Period | Current | RCP2.6 | | RCP6.0 | | RCP8.5 | |
| --- | --- | --- | --- | --- | --- | --- | --- | --- |
|  |  |  | 2050s | 2070s | 2050s | 2070s | 2050s | 2070s |
| *P. pratensis* | Low suitable zone | 28.10 | 28.70 | 28.98 | 28.95 | 27.94 | 28.23 | 28.61 |
|  | Medium suitable zone | 15.96 | 15.89 | 15.73 | 16.09 | 15.98 | 15.93 | 15.75 |
|  | High suitable zone | 3.69 | 3.20 | 3.10 | 3.29 | 3.80 | 3.79 | 3.68 |
|  | Total suitable zone | 47.75 | 47.78 | 47.81 | 48.33 | 47.72 | 47.94 | 48.04 |
| *P. araratica* | Low suitable zone | 28.02 | 40.06 | 36.99 | 41.05 | 43.11 | 42.89 | 49.08 |
|  | Medium suitable zone | 16.20 | 24.27 | 23.12 | 26.05 | 30.13 | 28.02 | 36.53 |
|  | High suitable zone | 3.87 | 7.15 | 8.08 | 8.20 | 12.36 | 12.03 | 18.54 |
|  | Total suitable zone | 48.09 | 71.48 | 68.19 | 75.31 | 85.60 | 82.95 | 104.15 |
| *P. pratensis var. anceps* | Low suitable zone | 16.21 | 21.33 | 20.41 | 22.06 | 24.48 | 22.40 | 25.93 |
|  | Medium suitable zone | 6.61 | 7.39 | 7.29 | 7.50 | 7.42 | 7.00 | 7.23 |
|  | High suitable zone | 2.37 | 2.90 | 2.72 | 2.98 | 3.47 | 3.38 | 3.64 |
|  | Total suitable zone | 25.19 | 31.62 | 30.42 | 32.53 | 35.38 | 32.78 | 36.81 |
| *F. sinensis* | Low suitable zone | 20.40 | 20.79 | 20.47 | 20.36 | 20.75 | 17.23 | 17.19 |
|  | Medium suitable zone | 7.77 | 7.40 | 7.50 | 7.07 | 7.31 | 6.88 | 7.10 |
|  | High suitable zone | 3.03 | 3.41 | 2.85 | 2.71 | 3.08 | 3.14 | 3.03 |
|  | Total suitable zone | 31.20 | 31.60 | 30.82 | 30.15 | 31.14 | 27.25 | 27.31 |
| *E. nutans* | Low suitable zone | 47.64 | 64.87 | 64.45 | 69.95 | 76.19 | 77.57 | 87.62 |
|  | Medium suitable zone | 24.76 | 31.70 | 31.49 | 34.32 | 39.34 | 38.00 | 47.25 |
|  | High suitable zone | 8.32 | 12.50 | 12.52 | 13.43 | 15.75 | 14.51 | 17.61 |
|  | Total suitable zone | 80.73 | 109.06 | 108.46 | 117.71 | 131.28 | 130.08 | 152.49 |
| *L. perenne* | Low suitable zone | 12.44 | 16.24 | 15.85 | 16.74 | 18.28 | 18.96 | 21.41 |
|  | Medium suitable zone | 3.44 | 4.52 | 4.18 | 4.76 | 4.99 | 5.06 | 5.86 |
|  | High suitable zone | 0.81 | 1.14 | 0.94 | 1.09 | 1.26 | 1.32 | 1.60 |
|  | Total suitable zone | 16.69 | 21.89 | 20.98 | 22.60 | 24.54 | 25.34 | 28.88 |

**Table S3:** Centroid coordinates of typical vegetation restoration species on the QTP.

| Period | Latitude and Longitude | *P*. pratensis | *P*. araratica | *P. pratensis* var. *anceps* | *F. sinensis* | *E. nutans* | *L. perenne* |
| --- | --- | --- | --- | --- | --- | --- | --- |
| current | Longitude (°E) | 97.444 | 98.028 | 98.676 | 100.933 | 95.986 | 98.589 |
|  | Latitude (°N) | 31.146 | 32.187 | 32.422 | 34.751 | 31.809 | 30.671 |
| RCP2.6 2050s | Longitude (°E) | 97.204 | 97.368 | 98.265 | 98.865 | 95.759 | 98.592 |
|  | Latitude (°N) | 31.135 | 32.019 | 32.655 | 32.901 | 32.080 | 30.838 |
| RCP2.6 2070s | Longitude (°E) | 97.195 | 97.679 | 98.433 | 99.077 | 95.701 | 98.451 |
|  | Latitude (°N) | 31.158 | 32.168 | 32.685 | 33.130 | 32.041 | 30.851 |
| RCP6.0 2050s | Longitude (°E) | 97.184 | 97.378 | 98.194 | 98.761 | 95.734 | 98.543 |
|  | Latitude (°N) | 31.177 | 32.067 | 32.666 | 33.012 | 32.149 | 30.891 |
| RCP6.0 2070s | Longitude (°E) | 97.250 | 97.066 | 98.231 | 98.911 | 95.834 | 98.394 |
|  | Latitude (°N) | 31.161 | 32.142 | 32.881 | 33.052 | 32.233 | 30.952 |
| RCP8.5 2050s | Longitude (°E) | 97.125 | 97.057 | 98.284 | 98.972 | 95.704 | 98.392 |
|  | Latitude (°N) | 31.206 | 32.213 | 32.942 | 33.312 | 32.230 | 30.991 |
| RCP8.5 2070s | Longitude (°E) | 97.021 | 96.240 | 98.223 | 98.942 | 95.709 | 98.260 |
|  | Latitude (°N) | 31.256 | 32.163 | 33.132 | 33.351 | 32.243 | 31.230 |

**Table S4:Centroid migration distances of species under different climate scenarios**

| Species | RCP scenarios | Current-2050s/km | 2050s-2070s/km | Current-2070s/km |
| --- | --- | --- | --- | --- |
| *P*. *pratensis* | RCP2.6 | 22.87 | 2.7 | 25.57 |
|  | RCP6.0 | 24.98 | 6.53 | 31.5 |
|  | RCP8.5 | 31.07 | 11.34 | 42.42 |
| *P*. *araratica* | RCP2.6 | 64.91 | 33.66 | 98.57 |
|  | RCP6.0 | 62.65 | 30.55 | 93.19 |
|  | RCP8.5 | 91.41 | 77.08 | 168.49 |
| *P*. *pratensis* var*. anceps* | RCP2.6 | 46.43 | 16.08 | 62.5 |
|  | RCP6.0 | 52.7 | 24.16 | 76.86 |
|  | RCP8.5 | 68.48 | 21.88 | 90.36 |
| *F*. *sinensis* | RCP2.6 | 280.71 | 32.24 | 312.95 |
|  | RCP6.0 | 278.54 | 14.67 | 293.21 |
|  | RCP8.5 | 241.36 | 5.16 | 246.51 |
| *E*. *nutans* | RCP2.6 | 36.97 | 6.98 | 43.95 |
|  | RCP6.0 | 44.66 | 13.26 | 57.92 |
|  | RCP8.5 | 53.84 | 1.52 | 55.36 |
| *L*. *perenne* | RCP2.6 | 18.57 | 13.54 | 32.11 |
|  | RCP6.0 | 24.85 | 15.75 | 40.6 |
|  | RCP8.5 | 40.25 | 29.4 | 69.65 |
